# Magnetic Moments in Single-Atom Alloys: Trends, Mechanisms, and Implications for Tunable Adsorption

**DOI:** 10.1021/acsphyschemau.6c00086

**Published:** 2026-08-18

**Authors:** Shengjie Zhang, Collette I. Riviere, Matthew M. Montemore

**Affiliations:** † Department of Chemical and Biomolecular Engineering, 5783 Tulane University, New Orleans, Louisiana 70118, United States; ‡ Hangzhou Yanqu Information Technology Co. Ltd. Hangzhou 310003, P. R. China

**Keywords:** single-atom alloys, magnetic moments, density functional theory, hydrogen adsorption, Stoner criterion, d-band model, heterogeneous catalysis

## Abstract

Single-atom alloys have proven to have promising and unusual catalytic, chemical, and electronic structure properties. However, the mechanism underlying their magnetic states and the effect of these states on their chemical and catalytic properties are not well understood, despite the demonstrated importance of magnetism in catalysis. Here, we study the magnetic states of single-atom alloys consisting of transition metals embedded in Cu, Ag, and Au hosts. We find that many single-atom alloys feature strong magnetic moments, associated with large energetic differences between spin-polarized and nonspin-polarized states as well as large effects on adsorption energies. Indeed, the magnetic moment linearly correlates with the effect of the spin on adsorption. The Stoner criterion is generally quite accurate in predicting whether magnetism occurs. The shift of the d-band center roughly correlates with the change in the adsorption energy upon inclusion of spin, although changes in the charge on the dopant atom also play a role for Ag-host single-atom alloys. This work opens pathways to leverage magnetic properties in single-atom alloys, including by the static modification of reactivity, dynamically programming energetics, or local magnetic heating at the single-atom site.

## Graphical abstract

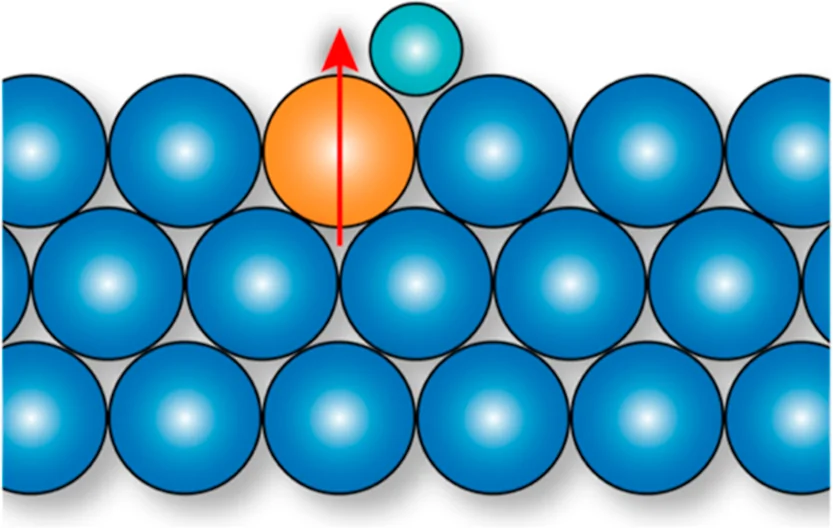

## Introduction

Single-atom alloys (SAAs) have received significant research attention due to their chemical and catalytic properties.^1–5^ SAAs consist of isolated atoms of a dopant metal embedded in the surface of a host metal. Past studies have shown that they can be highly efficient and selective catalysts for a variety of reactions. More fundamentally, they can break adsorption-energy scaling relations and Brønsted–Evans–Polanyi relations,^6–10^ and they can exhibit spillover.^1,11,12^ These chemical properties allow them to facilitate initial bond activation steps without binding downstream intermediates too strongly, which is highly desirable for many catalytic reactions. Finally, SAAs have shown interesting and unusual electronic structure properties^13–15^ and share features with both more traditional metal alloys as well as supported single-atom catalysts. However, a comprehensive study of their magnetic properties and how they affect their chemical reactivity is lacking.

Spin and magnetism have proven to have significant impacts on the catalytic performance of magnetic metals and oxides, as well as energetics for adsorption and reaction.^16–30^ The spin state can be influenced in experiments by external fields or by modifying the material (e.g., alloying) or in computation by artificially modifying the spin state. Magnetic effects can be particularly pronounced for reactants that are spin-polarized, such as O_2_.^31–33^ Spin typically destabilizes adsorption, and the effect of spin has been rationalized in terms of the d-band model.^34,35^

Furthermore, single-atom catalysts and nanoclusters can display large effects of spin on the chemical properties. For example, modifying the magnetic state of single-atom catalysts can affect their electrocatalytic performance.^36,37^ Similarly, spin and spin transitions in nanoclusters can greatly affect their chemical reactivity toward molecules.^38^

Previous studies indicate that SAAs can display magnetic moments even in cases where both the host and dopant are nonmagnetic in their pure forms.^13,39^ Indeed, isolated dopants have long been known to induce magnetic moments in some bulk solids.^40^ Additionally, SAAs can display unusual electronic structure in the form of so-called free-atom-like d states, where the d electrons are localized at the dopant site and exhibit a sharp peak in the density of states.^13–15^ Thus, it is important to properly consider the spin state when studying SAAs in order to properly capture their chemical and catalytic properties. Furthermore, it may be possible to leverage the spin-state of single-atom alloys using static or dynamic external fields to modify reactivity, dynamically program catalytic behavior,^41^ or magnetically heat the single-atom active site.^42,43^ Thus, a careful elucidation of the magnetic state of SAAs and its effect on the surface chemical properties is needed.

Recent work has also begun to establish systematic connections among the electronic structure, magnetism, and adsorption properties of SAAs. For example, spin-polarized and nonspin-polarized calculations have been compared across a broad range of SAAs, resulting in periodic trends in both magnetic moments and adsorption energetics. A molecular orbital framework was proposed, closely related to electron-counting concepts such as the 10-electron rule, to rationalize how dopant-host interactions govern magnetic behavior and adsorbate binding.^44^ These studies highlight that magnetism and adsorption are intimately connected manifestations of the underlying electronic structure of SAAs. However, a systematic assessment of the magnitude of magnetic stabilization across SAA families and of how magnetism quantitatively modifies adsorption energetics remains limited.

Here, we study the magnetic states of SAAs with Cu, Ag, and Au as hosts. We first establish trends in magnetic moments across these SAAs, finding that many display large magnetic moments with significant energetic differences between nonmagnetic and magnetic states. We then assessed the predictive power of the Stoner criterion, which proved to be quite accurate for identifying the presence of magnetism. Next, we examine how magnetism modifies adsorption energetics, showing that magnetism consistently weakens adsorption and that this effect can be rationalized primarily through shifts in the d-band center. Our results indicate that magnetism is an important and underexplored dimension of SAA behavior with implications for both accurate computational modeling and the potential tuning of reactivity through magnetic effects.

## Methods

Density functional theory (DFT) calculations were carried out using the Vienna Ab initio Simulation Package (VASP) with a plane-wave basis set and projector-augmented wave (PAW) pseudopotentials.^45,46^ Exchange–correlation effects were treated with the Perdew–Burke–Ernzerhof (PBE) functional, and van der Waals interactions were accounted for with the Tkatchenko–Scheffler method.^47^ A 7 × 7 × 1 *k*-point mesh was applied to a 3 × 3 (111) surface cell consisting of four layers, with the bottom two layers fixed. The plane-wave energy cutoff was set to 400 eV. Methfessel–Paxton^48^ smearing of order 2 was used with a width of 0.2 eV. Our convergence test in our past work^13^ on similar systems suggests our computational parameters are sufficiently converged. Both spin-polarized and nonpolarized calculations were performed. Several initial magnetic configurations were tested in many cases by assigning different initial magnetic moments to the dopant atom, while assigning the host atoms no magnetic moment. All reported magnetic moments are total magnetic moments. We performed a test for Fe_1_Au where we projected the magnetic moment on each atom and found that Fe’s projected magnetic moment was within 0.01 μ_B_ of the total, and all other atoms had magnetic moments less than 0.01 μ_B_. Thus, while delocalized moments have been reported in Pd-host SAAs,^13,40^ we expect the systems studied here to feature magnetism highly localized on the dopant atom. Hydrogen adsorption energies were referenced to molecular H_2_ in the gas phase, with the convention that exothermic adsorption corresponds to negative values. Electronic self-consistency was achieved with a convergence threshold of 10^–5^ eV, and structural relaxations were performed until forces were below 0.03 eV/Å. Adsorbed H was positioned in the fcc hollow site and allowed to fully relax. Test calculations on strong-binding dopants indicate that the fcc hollow site is the most stable site except for Ir-doped SAAs, and in these cases the H spontaneously moves to the top site. All simulations used the lattice constants of the corresponding pure metals.

Spin–orbit coupling was omitted in this study. Table S3 shows the small effect of spin–orbit coupling on the magnetic moments of representative Au-host SAAs. Furthermore, dipole corrections were not included in the production calculations. To assess the possible influence of slab thickness and dipole corrections, we performed additional test calculations for representative systems, including Co_1_Ag, Fe_1_Au, and Mn_1_Ag (Table S4). Specifically, H adsorption energies were compared by using four-layer slabs, six-layer slabs, and four-layer slabs with dipole corrections. These tests showed that the resulting changes in the H adsorption energies were no larger than 0.02 eV. Therefore, all adsorption energies reported in this paper were calculated using four-layer slabs without dipole corrections.

For the denisty of states (DOS) analysis, calculations were carried out using a fixed charge density, a 15 × 15 × 1 *k*-point mesh, and 324 bands in total. The number of states at the Fermi energy was calculated using an average over a window of 0.05 eV above and below the Fermi energy. The d-band center included states up to 0.3 eV above the Fermi energy, as in previous work.^13^

Our calculations are mostly consistent with previous work where values are available, with some disagreement in some cases (e.g., we predict a nonzero magnetic moment for Co_1_Cu, in contrast to some previous work^49^). The variations likely occur mostly in cases where the energy difference between the spin-polarized and nonspin-polarized states is small. In these cases, changes in the computational setup may influence the preferred spin state, and it can be difficult to converge to the correct spin state in every case when performing a reasonably large number of calculations, both of which may lead to some disagreement across studies. For surfaces where spin has only a small effect on energetics, this disagreement in the spin state will be unlikely to have much effect on calculated adsorption and reaction energies, although it may still be important to capture the correct spin state if one is considering magnetic heating, valence band energies, or other properties that directly rely on the spin state.

## Results and Discussion

### Magnetic Moments and Energy Differences

DFT calculations were employed to determine the magnetic states of SAAs for d-block dopants embedded in the surfaces of Cu, Ag, and Au hosts. In agreement with expectations, elements such as Co, Cr, Mn, and Fe, which are generally magnetic in their pure forms, were predicted to exhibit magnetism when doped into Cu, Ag, and Au. A notable exception was Ni, which is magnetic in its pure state but was not found to exhibit magnetism when doped into any surface. The calculations also indicated magnetism for some dopants that are not magnetic in their pure form, such as V, Tc, and Re in Ag, Au, and Cu; Ti, Mo, and W in Ag and Au; Nb and Ta in Ag; and Ru in Au (Figure 1a).

**Figure 1:**
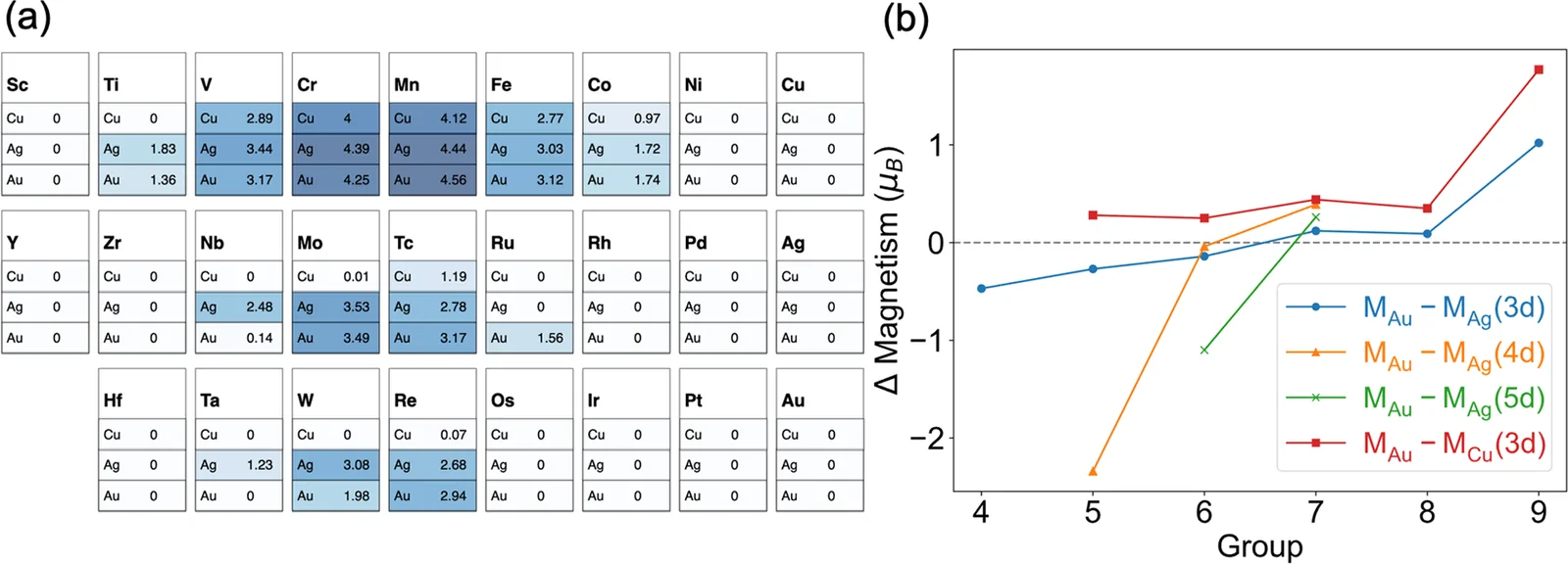
(a) Calculated magnetic moments of SAAs in μ_B_, with the host (Cu/Ag/Au) next to each value (groups 3–11). (b) Magnetic moment differences as a function of the dopant’s group.

As illustrated in Figure 1a, the highest magnetic moments were observed for dopants in the upper row (period 4, 3d elements) and the middle columns (groups 6 and 7). This trend can be explained by the larger spatial extent of the d orbitals for 4d and 5d dopants, which leads to stronger hybridization with the host orbitals and, consequently, to quenching of the magnetic moment. Additionally, the middle column elements exhibit the most unpaired electrons, contributing to enhanced magnetic behavior.

In addition to the dopant atom properties, the magnetic moments of SAAs are clearly influenced by the electronic structure of the host metal. The expansion of the d-band and contraction of the s-band in Au, due to relativistic effects, leads to a stronger s–d covalent admixture in Au as compared to Ag. This admixture shifts Au’s electronic bands toward the Fermi level, which enhances hybridization between Au’s s orbitals and the dopants’ d orbitals, ultimately leading to magnetic moment quenching. In contrast, Ag possesses a deeper d band, which allows it to preserve the atomic magnetism of the dopants in SAAs. Accordingly, since Cu features the highest d-band of the coinage metals and smallest lattice constant, we expect the magnetic moments to trend as Ag-host > Au-host > Cu-host. Indeed, Cu-host SAAs always feature the lowest magnetic moments, and the expected trend between Au and Ag holds true for early dopant elements, from group 4 to group 6.

Moving across a period, however, this ordering smoothly reverses: the moment difference between Ag and Au increases nearly monotonically and changes sign near group 7 for all three periods, so that for late dopants, the Au-host SAA retains the larger moment (Figure 1b). A similar upward trend occurs for Au versus Cu. Because this change is gradual and monotonic rather than peaked near the half-filled shell, it cannot be attributed to factors tied to d-band filling, such as the number of unpaired d electrons or d-electron energies, which would instead produce nonmonotonic, “V”-shaped trends with an extremum near groups 6–7. Instead, it tracks Janak’s distinction between the more “free-electron-like” spin density of early transition metals and the more “d-like” spin density of late transition metals.^50^ Two competing host effects underlie this behavior: the relativistic s–d band shift in Au enhances hybridization and quenches the moment, dominating for early dopants and keeping Au-host moments below Ag; but as late dopants become more “d-like” and localized, they are less susceptible to this quenching, so beyond group ∼7 Au-host SAAs retain more of their atomic moment than Ag-host SAAs.

To gain some insight into the spatial localization of the spin, we examined real-space spin-density distributions (Figure S5, isosurface = 0.004 e/Å^3^) for two cases. These distributions show that Fe_1_Au exhibits strongly anisotropic spin density with well-defined positive and negative lobes centered around the Fe dopant, consistent with a substantial contribution from directional Fe d orbitals and their hybridization with the surrounding Au atoms. In contrast, Ti_1_Au shows a more diffuse and approximately spherical spin-density distribution around Ti, without pronounced d-orbital-like angular features. The contrasting spatial distributions therefore suggest that the magnetism of Fe_1_Au is comparatively localized and d-like, whereas that of Ti_1_Au is more delocalized and consistent with a more free-electron-like contribution.

To quantitatively clarify the distinction between the “free-electron-like” behavior of early transition-metal SAAs and the “d-like” behavior of late-transition-metal SAAs, maximally localized Wannier functions were calculated for Ti_1_Au and Fe_1_Au. In Ti_1_Au, the Ti-centered Wannier functions exhibit relatively large average spreads of 1.86–2.46 Å^2^ and strong total hopping amplitudes of 22.17–25.97 eV, indicating spatially extended, itinerant states arising from strong Ti-d–Au-sp hybridization. By contrast, Fe_1_Au contains strongly localized Fe-d states, particularly in the majority-spin channel, with an average spread of only 0.70 Å^2^ and a total hopping amplitude of 11.99 eV. These quantitative Wannier descriptors support the physical picture that early transition-metal impurities in Au tend to form more spatially extended and itinerant, “free-electron-like” states, whereas late-transition-metal impurities retain more localized, atomic-d-like states. Recent work has further examined the periodic trends and confinement effects that govern when dopant d-states in SAAs retain free-atom-like character, providing additional support for the systematic early-to-late transition in the localized versus delocalized nature of the dopant states.^51^

The energy difference between the SAA with a magnetic moment (spin-polarized calculation) and without a magnetic moment (nonspin-polarized calculation) was calculated, as illustrated in Figure 2a. The energetic importance of correctly identifying the spin state varies from absolutely crucial (≈2 eV) to negligible (≈0.01 eV). Notably, this energy difference mirrors the trend observed in the magnetic moments, with the most pronounced differences occurring in the upper rows and middle columns. Such a trend is expected given that many theoretical models, including those by Anderson and Stoner, have treated the splitting between the spin-up and spin-down bands as proportional to the local magnetic moment, and consequently should be correlated to the energy difference.^52–55^

**Figure 2:**
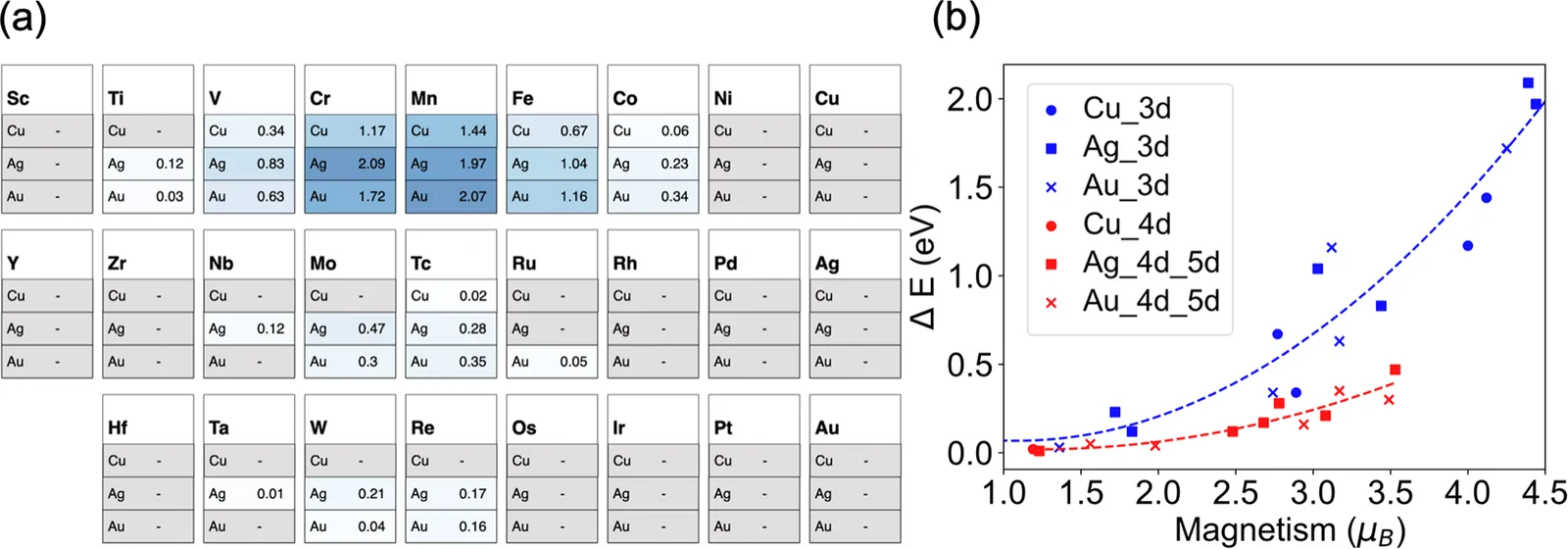
(a) Calculated energy difference between spin-polarized and nonspin-polarized SAAs (groups 3–11). (b) Relationship between the energy difference in (a) and the corresponding magnetic moment (with quadratic regression).

In Figures 2b and S1, we plot the calculated energy differences (from Figure 2a) against the magnetic moments (from Figure 1a). Rather than grouping them by host metal, we observe that dopants from period 4 (3d metals) can be categorized into one group, while dopants from periods 5 and 6 (4d and 5d metals) form another. Both groups can be reasonably well-described by linear fits (Figure S1). However, there appears to be some nonlinearity, and there is a noticeable intercept for the 3d metals, such that the energy difference predicted by this fit is nonzero even when the magnetic moment is absent. A quadratic fit yields an improved fit (Figure 2b). The approximations made in the Stoner model, from eqs S4 and S5, involve neglecting higher-order terms when expanding the exchange–correlation potential in terms of the magnetization density m(r⃗) and a density-dependent potential Ṽ(ρ(r⃗)) . Retaining only the first-order term results in a simplified model that provides a straightforward understanding of exchange–correlation potential splitting. However, the second-order term becomes significant when the magnetization m(r⃗) is large and cannot be treated as a small perturbation. Our calculations suggest that including the second-order term ${\left[m\left(\mathrm{r⃗}\right)\right]}^{2}Ṽ\left(\rho \left(\mathrm{r⃗}\right)\right)$ may enhance the model’s accuracy when dealing with the energy difference, particularly in cases where the magnetization is large. Furthermore, these results suggest that for magnetic moments less than roughly 1 μ_B_, the energy difference is essentially negligible. Thus, if some SAAs actually feature ground states with small magnetic moments, it may be difficult to correctly converge to this ground state, and an experimental sample could potentially feature dopant sites that fluctuate between magnetized and nonmagnetized states.

### Stoner Criterion Prediction

The Stoner criterion uses a simple model to predict when spontaneous magnetization should occur, with the final expression as

1 $$I\times D\left(E_{F}\right)>1$$

where the Stoner parameter *I* describes the strength of the exchange interaction among electrons, and *D*(*E*_F_) is the number of electronic states at the Fermi energy in the nonspin-polarized state. If the product of these values is greater than 1, the material is expected to exhibit spontaneous magnetism, and if the product is less than 1, the material is not expected to be spontaneously magnetic. Briefly, the Stoner model uses *IM* in eq S5, where *M* is the magnetization, to describe the exchange splitting, and $\pm \frac{1}{2}IM$ for a constant shift in the DOS (see the Supporting Information for more details). The resulting Stoner criterion was initially developed to predict the magnetism of pure metals and alloys. Later, Deutz^56^ demonstrated its applicability to predict the magnetism of impurities. As previously noted, the Stoner model considers only the first-order term of m(r⃗) , with higher-order terms neglected. Fortunately, eq S8 is a derivative of eq S7 at *M* = 0, which causes these terms to vanish and does not change the prediction accuracy. Janak calculated the Stoner parameter *I* for the metals from Li to In and found that it is less sensitive to the crystal structure than the DOS.^50^ Accordingly, we determined our Stoner criterion using the product of *I* from Janak’s work and *D*(*E*_F_) from our DFT calculations, as shown in Figure S2.

The Stoner criterion is accurate in most cases for predicting magnetism in SAAs (Figure 3). For Au and Ag, only three predictions are incorrect (Hf_1_Ag, Ru_1_Au, Ta_1_Au), all with Stoner criterion values between 0.9 and 1.1, close to the cutoff of 1 where lower accuracy may be expected, in part due to small moments being more sensitive to computational settings. Thus, classifications in this regime should be interpreted cautiously. For Cu, there are two additional incorrect predictions that are also near the cutoff, Mo_1_Cu and Ta_1_Cu. Notably, all of these dopants display magnetism in at least one host, except Hf (see Figure 1), suggesting that these incorrect predictions may be cases that are nearly magnetic. Indeed, for Mo_1_Cu, our calculations predict a very small, but nonzero, magnetic moment of 0.01 μ_B_. It is also plausible that Hf_1_Ag could be on the cusp of magnetism since the neighboring element Ta displays magnetism in Ag. Thus, the Stoner criterion is a useful first-order predictor for the vast majority of cases, with most discrepancies occurring near the threshold where borderline magnetism and/or numerical sensitivity is expected.

**Figure 3:**
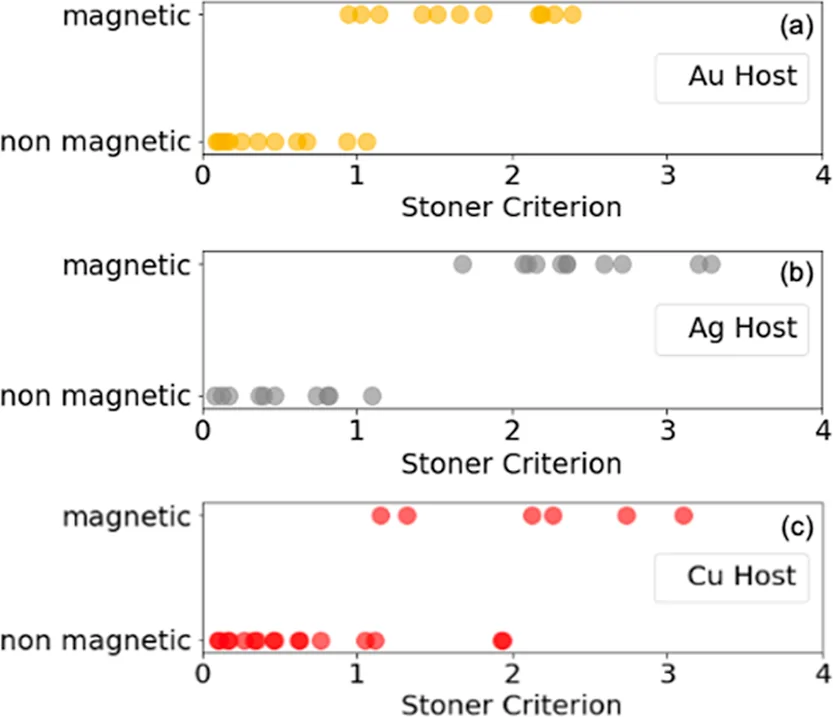
Magnetism of ground state (“magnetic” defined as a magnetic moment >0.10 μ_B_) vs Stoner criterion for SAAs. The cutoff of 1 is generally accurate in predicting the presence of magnetism.

Beyond the near-cutoff errors, there are two Cu-host SAAs that the Stoner criterion clearly predicts to be magnetic but appear nonmagnetic in our DFT calculations, Re_1_Cu and W_1_Cu. However, for Re_1_Cu, we in fact predict a small magnetic moment of 0.07 μ_B_, supporting the idea that the spin-polarized and nonspin-polarized states are very similar in energy, while W is magnetic in both Ag and Au. These observations suggest borderline magnetic behavior, even though their Stoner criterion values are not themselves borderline.

The accuracy of the Stoner criterion depends somewhat on the cutoff used to define magnetic vs nonmagnetic; with our cutoff of 0.10 μ_B_, the Stoner criterion achieves 90% accuracy, with 7% incorrect but near the Stoner criterion cutoff of 1 and only 3% representing significant outliers. Alternative cutoff choices shift which specific cases are outliers (potentially changing predictions for Nb_1_Au and Re_1_Cu) but do not substantially change the overall picture. Overall, the high accuracy of the Stoner criterion for predicting magnetism is somewhat surprising given the differences in electronic structure between SAAs and bulk metals, and suggests that magnetism in SAAs is governed by the similar local electronic structure features as in bulk metals. In addition to mechanistic insight, the Stoner criterion provides a practical tool for checking whether a given SAA is likely to exhibit magnetism, which may be particularly useful for cases that are difficult to converge to the correct magnetic state.

### Hydrogen Adsorption

We next examine the effect of magnetism on H adsorption, as a simple but important adsorbate. For pure metal surfaces, prior work has shown that adsorption is weaker on spin-polarized surfaces because the repulsive contribution from majority-spin states outweighs the attractive contribution from minority-spin states.^35^ Moreover, this effect holds across different adsorption sites and adsorbates.^34^ Our results confirm and extend this finding to SAAs.^44^ Specifically, adsorption on spin-polarized SAAs is systematically weaker than on nonspin-polarized counterparts. As shown in Figure 4a, we observe a qualitative trend: the degree of adsorption weakening grows with the SAA’s initial magnetism (maximum around first row, middle columns). The magnetism-induced weakening of adsorption (negative values in Figure 4a) reaches up to ≈0.33 eV, which is quite substantial for H adsorption, while in other SAAs, it can be nearly negligible (≈0.01 eV).

**Figure 4:**
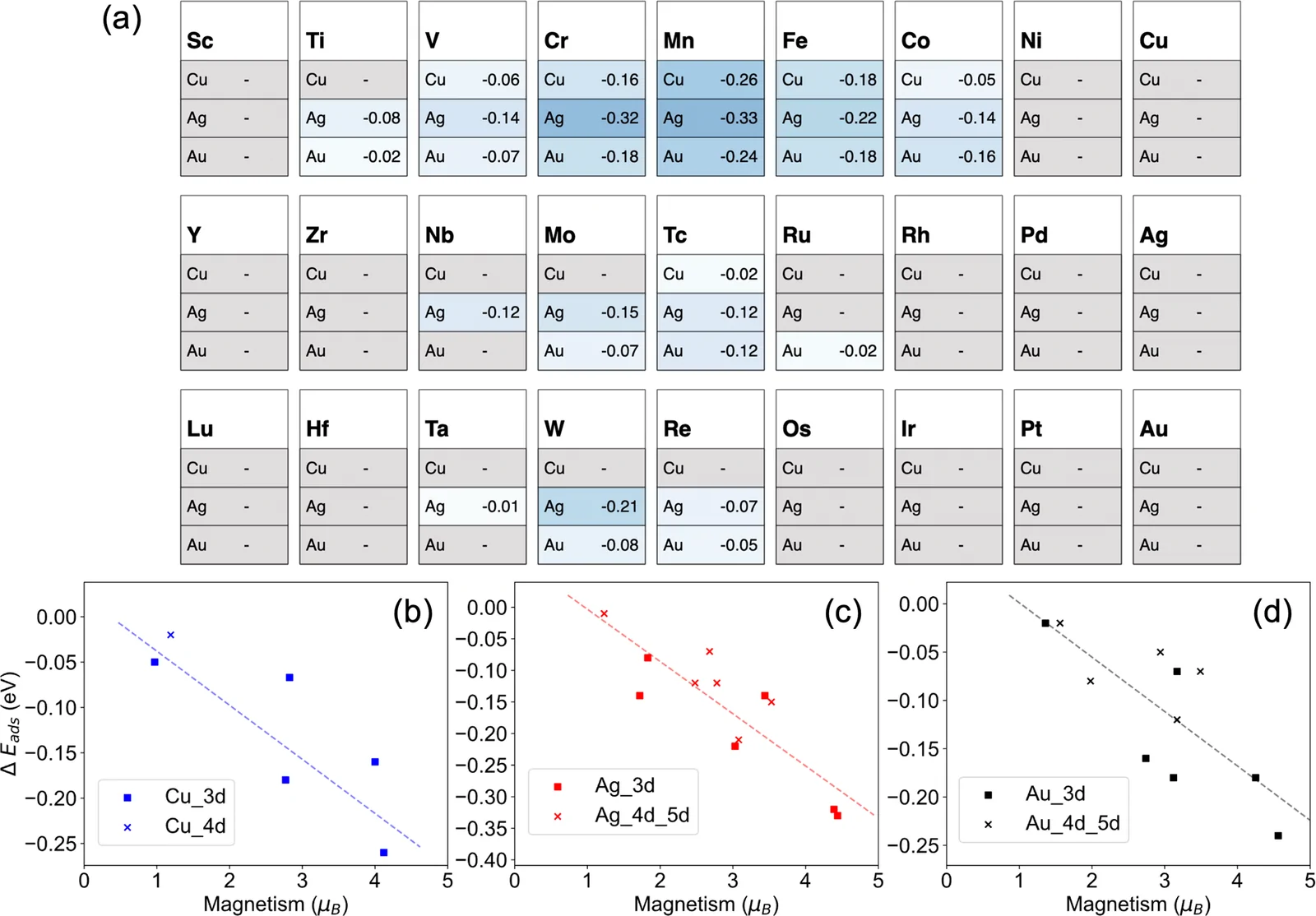
(a) Difference in H adsorption energy (eV) between the spin-polarized and nonspin-polarized cases, where a negative value indicates weaker adsorption on the spin-polarized surface (groups 3–11). (b–d) The same difference in H adsorption energy as a function of the magnetic moment, plotted separately for each host metal.

To test whether the spin-induced weakening of adsorption is specific to H or more generally relevant to molecular adsorbates, we additionally calculated CO adsorption on selected magnetic SAAs: Co_1_Ag, Co_1_Au, Fe_1_Ag, Fe_1_Au, Mn_1_Ag, and Mn_1_Au (Table S2). For each surface, both spin-polarized and nonspin-polarized calculations were performed. In all six cases, CO adsorption is weaker on the spin-polarized surface. This trend is consistent with the H adsorption results, where including spin polarization also systematically weakens adsorption. The larger spin effect observed for CO than for H in several of these systems may be related to the stronger interaction between the CO frontier orbitals and the dopant d states. Importantly, the CO results indicate that the spin-induced weakening of adsorption is not unique to H but can also occur for a molecular adsorbate with a different bonding motif, consistent with previous work.^34,35^ These results suggest that magnetism may be an important factor to consider when evaluating adsorption trends on SAAs, particularly for dopants with large local magnetic moments.

Furthermore, we find a rough linear correlation between the adsorption energy difference and the preadsorption magnetic moments (Figure 4b–d). The plots indicate that a quadratic regression would not provide an obviously better fit, in contrast to the observation shown in Figure 2b. We primarily attribute this to the fact that H adsorption energies involve a subtraction of the bare-surface and H-adsorbed systems, with energetic variations partially canceling and resulting in a simpler trend. The fact that H nearly always resides in a hollow site also moderates the trends and results in somewhat different trends for different host metals.

Adsorption on magnetic SAAs consistently reduces the magnetic moment of the dopant, as the adsorbate–surface interaction partially quenches the exchange splitting of the d electrons. In a magnetic SAA, the dopant’s unpaired d-electrons contribute to the system’s magnetization via the exchange interaction, which shifts spin-up states to higher energy and spin-down states to lower energy. Upon H adsorption, the electron density is redistributed within the dopant-host ensemble (rather than being transferred to a single atom), consistent with recent theoretical descriptions of adsorption-induced charge rearrangement in SAAs, including Ag-host systems.^57^ This redistribution changes the d-state occupation and partially screens the exchange interaction, reducing the spin-up/spin-down splitting and thus the dopant magnetic moment. Our calculations confirm this picture: every magnetic SAA loses some of its magnetic moment upon H adsorption (Figure S3). Specifically, the moment typically drops by approximately 0.5 μ_B_ for Ag- and Au-host SAAs, and by 0.25 μ_B_ for Cu-host SAAs. A simplistic picture would predict that the H atom’s electron would pair off with a dopant electron, reducing the spin by 1 μ_B_; however, the adsorbate–surface bonding is more delocalized and complex, and the partial screening of the exchange interaction described above results in a smaller change in spin.

### Factors Underlying Adsorption Energy Shifts

To connect the observed magnetic-adsorption trends to a unified electronic-structure framework, we turn to the d-band model. The original Hammer–Nørskov model correlates chemisorption strength with the position of a pure metal’s spin-averaged d-band center (ε_d_). Subsequent extensions by Nørskov and by Bhattacharjee introduce separate spin-up and spin-down d-band centers for spin-polarized metal surfaces.^34,35^ While the d-band model is a useful tool, it should be noted that SAAs have been known to deviate from simple d-band models, particularly for hydrogen adsorption,^58^ and in general the simplest form of the d-band model often does not give high quantitative accuracy.^59,60^

We start with the simplest model, which contains only Δε_d_ between spin-polarized and nonspin-polarized SAAs and correlates this with the corresponding change in hydrogen adsorption energy Δ*E*_ads_. Fitting Δ*E*_ads_ as a function of Δε_d_ (Figure 5a and Table S1) gives moderate predictive accuracy (*R* ^2^ = 0.55), with a coefficient of −0.11 for the change in d-band center and −0.06 eV for the intercept, indicating that the simplest d-band model can roughly capture the trends in Δ*E*_ads_. This model is in fact quite accurate for Cu- and Au-host SAAs but is fairly inaccurate for Ag-host SAAs.

**Figure 5:**
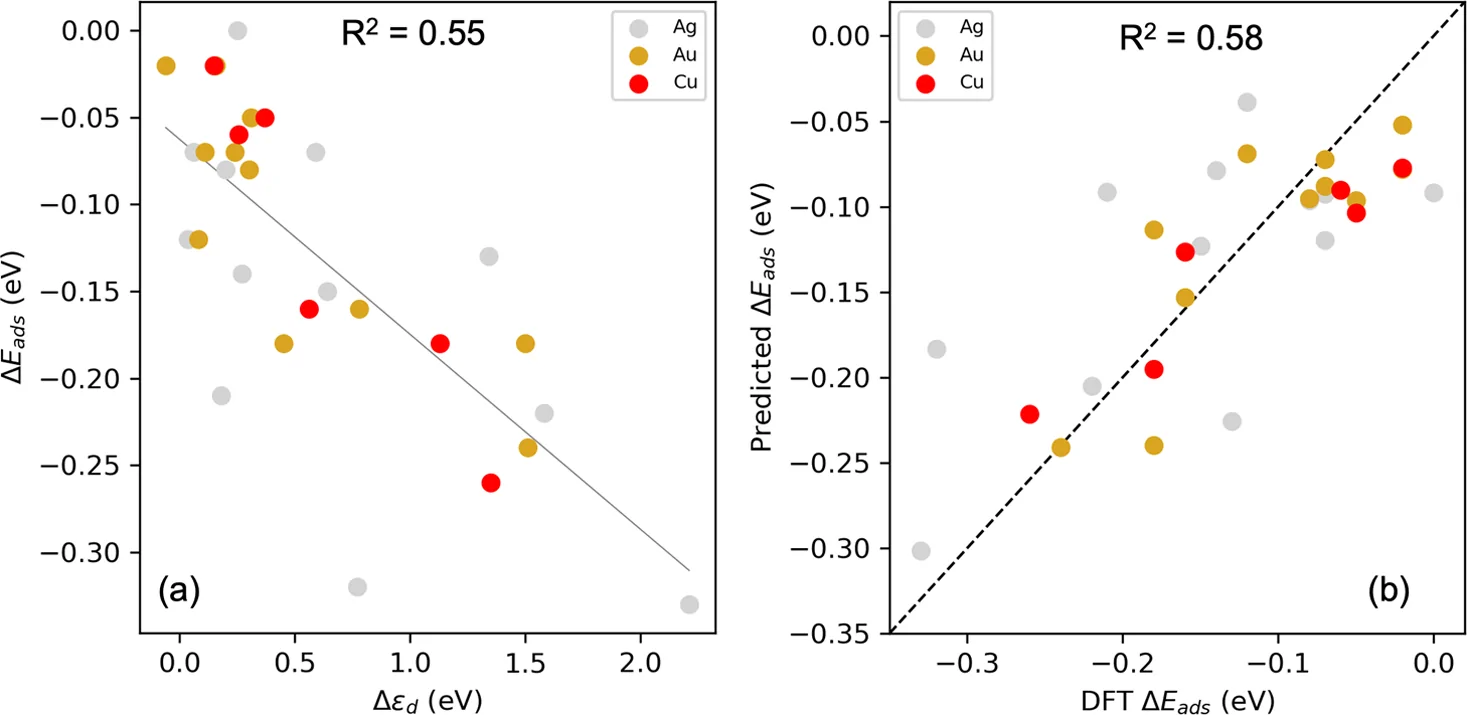
(a) Change in H adsorption energy Δ*E*_ads_ between spin-polarized and nonspin-polarized calculations as a function of the corresponding shift in the d-band center Δε_d_. *R* ^2^ values for each host metal are 0.37 (Ag), 0.67 (Au), 0.75 (Cu). (b) Parity plot comparing the DFT values and values predicted by a regression model with Δε_d_ and the dopant d-band charge change Δ*q*_d_, only for Ag-host SAAs. *R* ^2^ values for each host metal are 0.41 (Ag), 0.67 (Au), 0.77 (Cu).

Thus, for Ag-host SAAs, the d-band center shift alone is insufficient to accurately capture the change in adsorption energy, suggesting that additional electronic structure effects play a role. We found that the change in d-band charge on the dopant (Δ*q*_d_), similar to previous work,^61^ improves accuracy for Ag. This observation is consistent with prior analyses, which demonstrated that atomic charges can serve as useful descriptors for adsorption energetics in SAAs.^57^ Physically, Δ*q*_d_ reflects changes in the dopant d-state occupation. Within d-band theory, adsorption is influenced not only by the energy of the metal d states but also by their occupancy, since occupancy affects the filling of adsorbate–metal antibonding states.^62^ Consequently, systems with similar Δε_d_ may still exhibit different adsorption energies if spin-induced charge redistribution alters the d-state occupation. Test calculations indicated that this factor did not improve accuracy for Cu- and Au-host SAAs. A model incorporating Δ*q*_d_ for Ag-host surfaces alongside Δε_d_ for all hosts achieves an *R* ^2^ of 0.58 (Figure 5b), which improves further to 0.62 (Figure S4) when separate d-band center slopes are used for each host metal. These improvements are primarily driven by the Ag-host SAAs, which improve their *R* ^2^ from the quite poor 0.37 to a somewhat improved 0.41 between the two models in Figure 5, with only one additional fitting parameter. While the inclusion of d-band charge for Ag-host surfaces improved the model’s predictive power, the correlation (0.58) is not strong enough to comprehensively capture the quantitative details, indicating the influence of other additional electronic structure effects.

Our interpretation can also be viewed in the context of the molecular orbital framework recently proposed in the context of an electron-counting picture.^44^ In that picture, magnetism and adsorption trends arise from the occupancy and hybridization of localized dopant-centered states, with electron-counting concepts providing a useful organizing principle across the periodic table. From this perspective, the adsorption energy shifts observed here originate from spin-polarization-induced modifications of the electronic states involved in adsorbate bonding. While our analysis is formulated in terms of d-band descriptors, both approaches emphasize that changes in the electronic structure of the dopant underlie the coupling between the magnetism and adsorption. The improved performance obtained by including descriptors beyond the d-band center further suggests that factors related to orbital occupancy and charge redistribution can contribute to the adsorption trends in some SAAs.

## Conclusions

We have shown that many SAAs can feature magnetic ground states, even when both the host and dopant are nonmagnetic in their pure forms. A larger magnetic moment correlates with a larger difference in energy between the spin-polarized and nonspin-polarized states, as well as the degree of weakening of H adsorption. H adsorption in turn decreases the total spin, typically by 0.25 to 0.5 μB. This is less than the integer change expected from simple spin-pairing, reflecting the more delocalized nature of adsorbate–surface bonding. The Stoner criterion was found to be quite accurate for predicting magnetism in SAAs, particularly for Ag and Au hosts, suggesting that magnetism in SAAs is governed by similar local electronic structure features as in bulk metals. Overall, these results suggest that SAAs may behave more like pure metals than molecular species in terms of their magnetic properties.

These findings point to several opportunities for leveraging the spin in SAA catalysis. The complex electronic interactions between magnetic SAAs and adsorbates, involving partial screening of exchange splitting, d-band center shifts, and charge redistribution, may provide an additional lever for breaking linear scaling relations. Furthermore, the large, localized magnetic moment at the single-atom site makes many of these systems promising candidates for magnetic heating at the active site or for dynamically switching adsorption and reaction energetics via external fields, offering routes to tunable catalytic behavior beyond conventional alloying strategies.

## Supplementary Material



## Notes

Derivation of the Stoner criterion; energy difference versus magnetic moment; exchange integrals, densities of states at the Fermi level, and Stoner criteria; magnetic moments with and without adsorbed hydrogen; d-band-center and d-charge regression fits; real-space spin-density distributions of Ti_1_Au and Fe_1_Au; changes in adsorption energy, d-band center, and d-charge; CO adsorption energies; spin–orbit-coupling test; and slab thickness and dipole-correction tests (PDF)
