# Supplementary material for: Magnetic Moments in Single-Atom Alloys: Trends, Mechanisms, and Implications for Tunable Adsorption
Source: ACS Phys Chem Au. 2026 Aug 18;6(5):1274–82. doi: 10.1021/acsphyschemau.6c00086 (PMC13614053; doi:10.1021/acsphyschemau.6c00086)
Supplement: Supplementary file 1 [file pg6c00086_si_001.pdf]

Supporting Information for:

Magnetic Moments in Single-Atom Alloys: Trends, Mechanisms, and Implications for Tunable Adsorption

Shengjie Zhang,[a,b,†] Collette I. Riviere,[a,†] Matthew M. Montemore,*[,a]

[a]Department of Chemical and Biomolecular Engineering, Tulane University, New Orleans, Louisiana 70118, United States

[b]Hangzhou Yanqu Information Technology Co. Ltd. Hangzhou 310003, P. R. China

Email: mmontemore@tulane.edu

[†]These authors contributed equally

Equations for Stoner Criterion

In DFT calculations (LDA), the exchange-correlation potential $V_{xc}(\vec{r})$ is defined as

$$V_{xc}(\vec{r}) = \frac{\delta E_{xc}[\rho(\vec{r})]}{\delta\rho(\vec{r})} \quad , \tag{S1}$$

where $E_{xc}[\rho(\vec{r})]$ is the exchange-correlation energy, $\rho(\vec{r})$ is the electron density. When spin polarization is considered, the electron density $\rho(\vec{r})$ and magnetization density $m(\vec{r})$ can be described as

$$\rho(\vec{r}) = \rho^{+}(\vec{r}) + \rho^{-}(\vec{r}) \quad , \tag{S2}$$

$$m(\vec{r}) = \rho^{+}(\vec{r}) - \rho^{-}(\vec{r}) \quad , \tag{S3}$$

where $\rho^{+}(\vec{r})$ and $\rho^{-}(\vec{r})$ are spin-up and spin-down density. Considering $m(\vec{r})$ is a smaller parameter than $\rho(\vec{r})$, some models expand $V_{xc}(\vec{r})$ in terms of $m(\vec{r})$:

$$V_{xc}(\vec{r}) = V_{xc}^{0}(\vec{r}) \mp m(\vec{r})\tilde{V}\big(\rho(\vec{r})\big) \mp [m(\vec{r})]^{2}\tilde{V}\big(\rho(\vec{r})\big) \mp \cdots \quad , \tag{S4}$$

where $V_{xc}^{0}(\vec{r})$ is non-spin-polarized exchange-correlation potential. The Stoner model further approximates it as

$$V_{xc}(\vec{r}) = V_{xc}^{0}(\vec{r}) \mp \frac{1}{2} I \int m(\vec{r})\, d\vec{r} \quad , \tag{S5}$$

where *I* is the Stoner parameter, and $\int m(\vec{r})\, d\vec{r} = M$ is the integral of $m(\vec{r})$ over the unit cell. With these approximations, it can be expected that the splitting of the exchange-correlation potential, and consequently the energy difference, is proportional to the local magnetic moment *M*

$$[V_{xc}(\vec{r}) - V_{xc}^{0}(\vec{r})] \propto M \quad . \tag{S6}$$

The local magnetic moment M is obtained by

$$M = F(M) = \int^{E_F} [D\left(E + \frac{1}{2} IM\right) - D(E - \frac{1}{2} IM)]\, dE \quad , \tag{S7}$$

where E_F is the Fermi energy indicating an integration from the lowest to the Fermi level, *D(E)* is the density of states under non-spin polarization. To achieve magnetism, a non-zero solution must exist for $M = F(M)$. Eq (S7) is further simplified by taking the derivative at M = 0:

$$F'(0) = I * D(E_F) \quad . \tag{S8}$$

To have non-zero solutions for Eq (S7), Eq (S8) needs to be greater than 1. Thus,

$$I * D(E_F) > 1 \quad , \tag{S9}$$

also known as the Stoner criterion, is used to predict the spontaneous magnetization of a material.

Additional Results

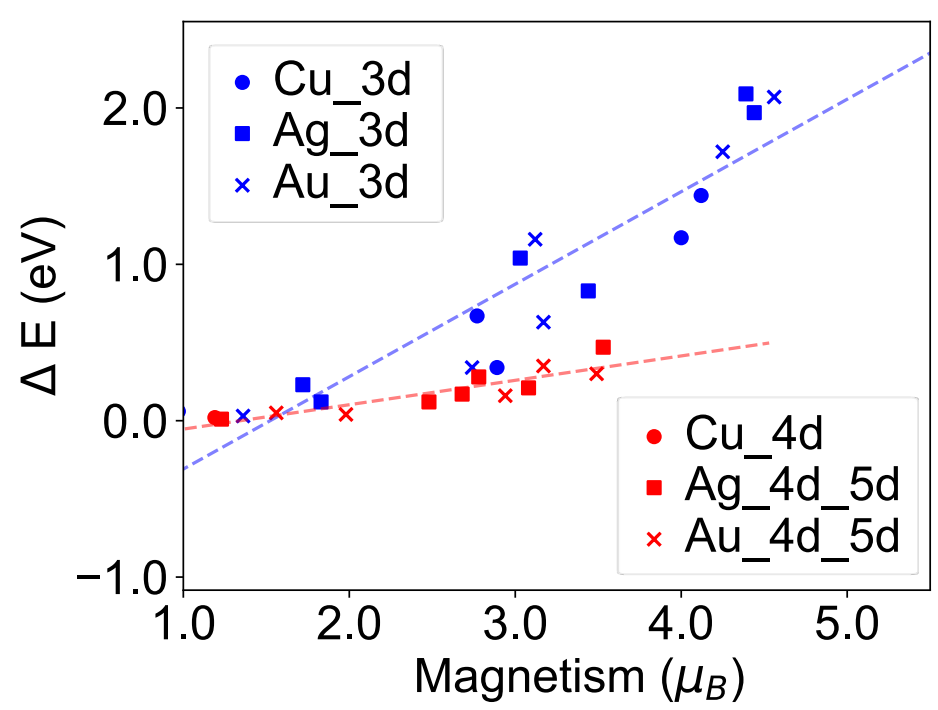

Figure S1. Difference in total energy between magnetic and non-magnetic states as a function of the magnetic state's total spin, with linear fit.

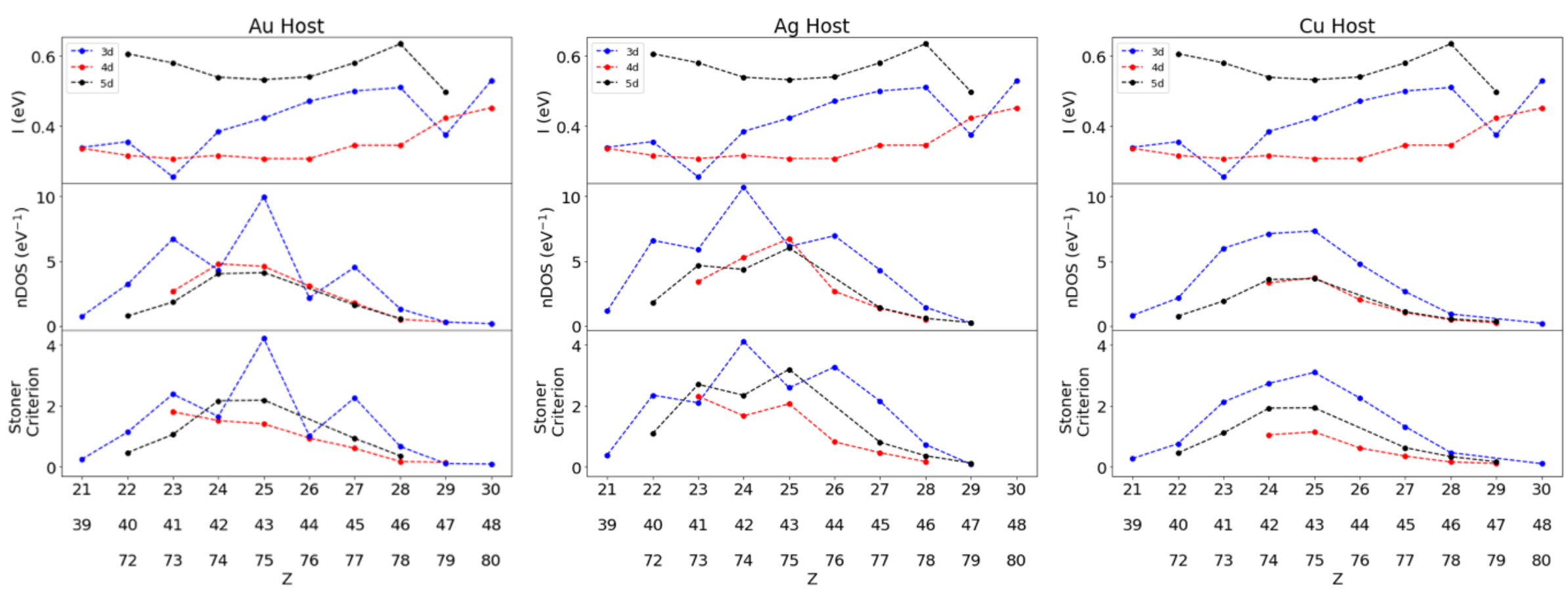

Figure S2. Exchange integral, density of states at the Fermi energy, and Stoner Criterion for magnetic SAAs in Au, Ag, and Cu host surfaces.

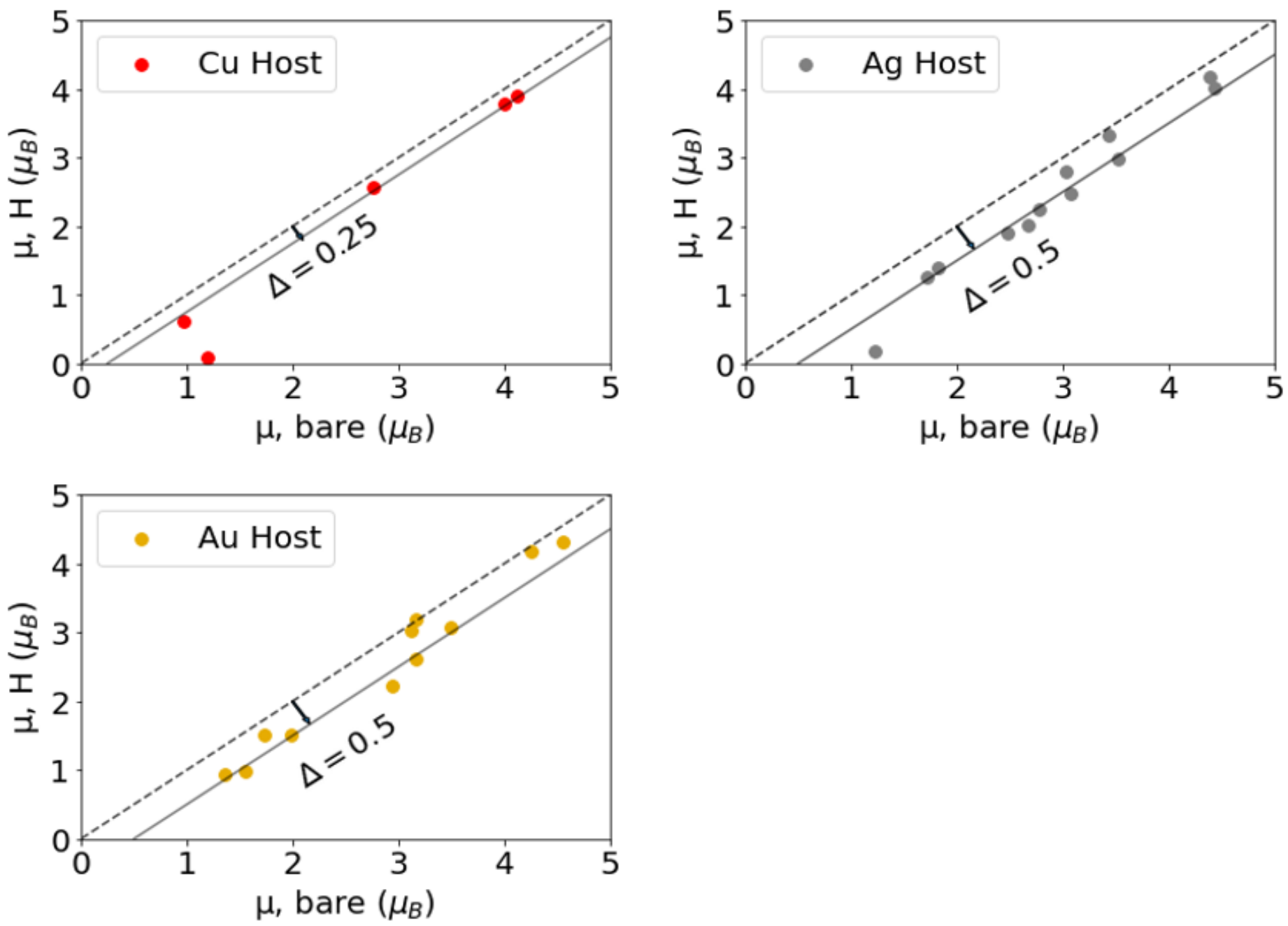

Figure S3. Magnetic moment with and without adsorbed H, along with lines corresponding to a given change in magnetic moment upon adsorption.

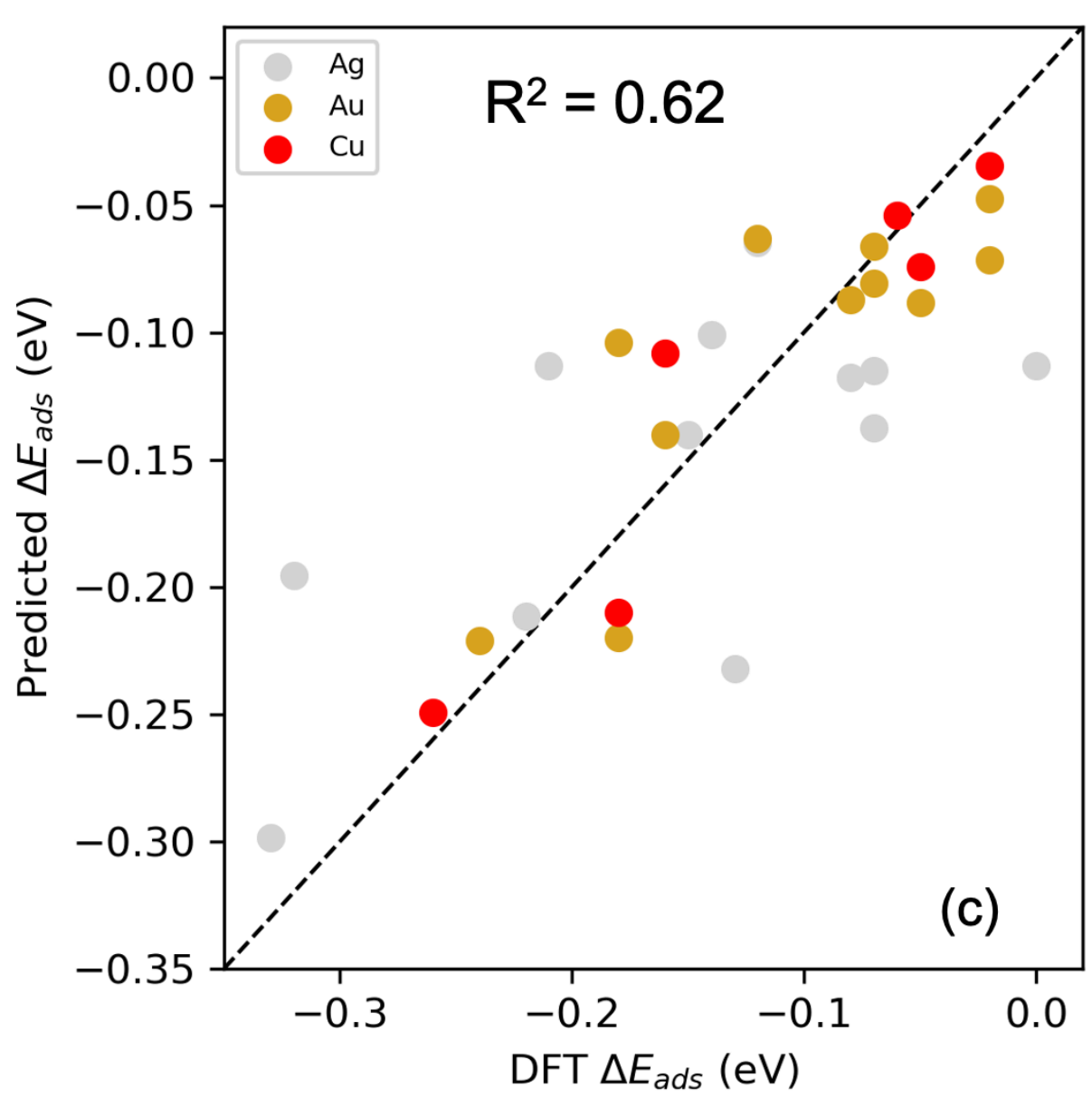

Figure S4. Parity plot comparing the DFT values and values predicted by a regression model with $\Delta\varepsilon_d$, fit separately for each host metal, and the dopant d-band charge change Δq_d, only for Ag-host SAAs.

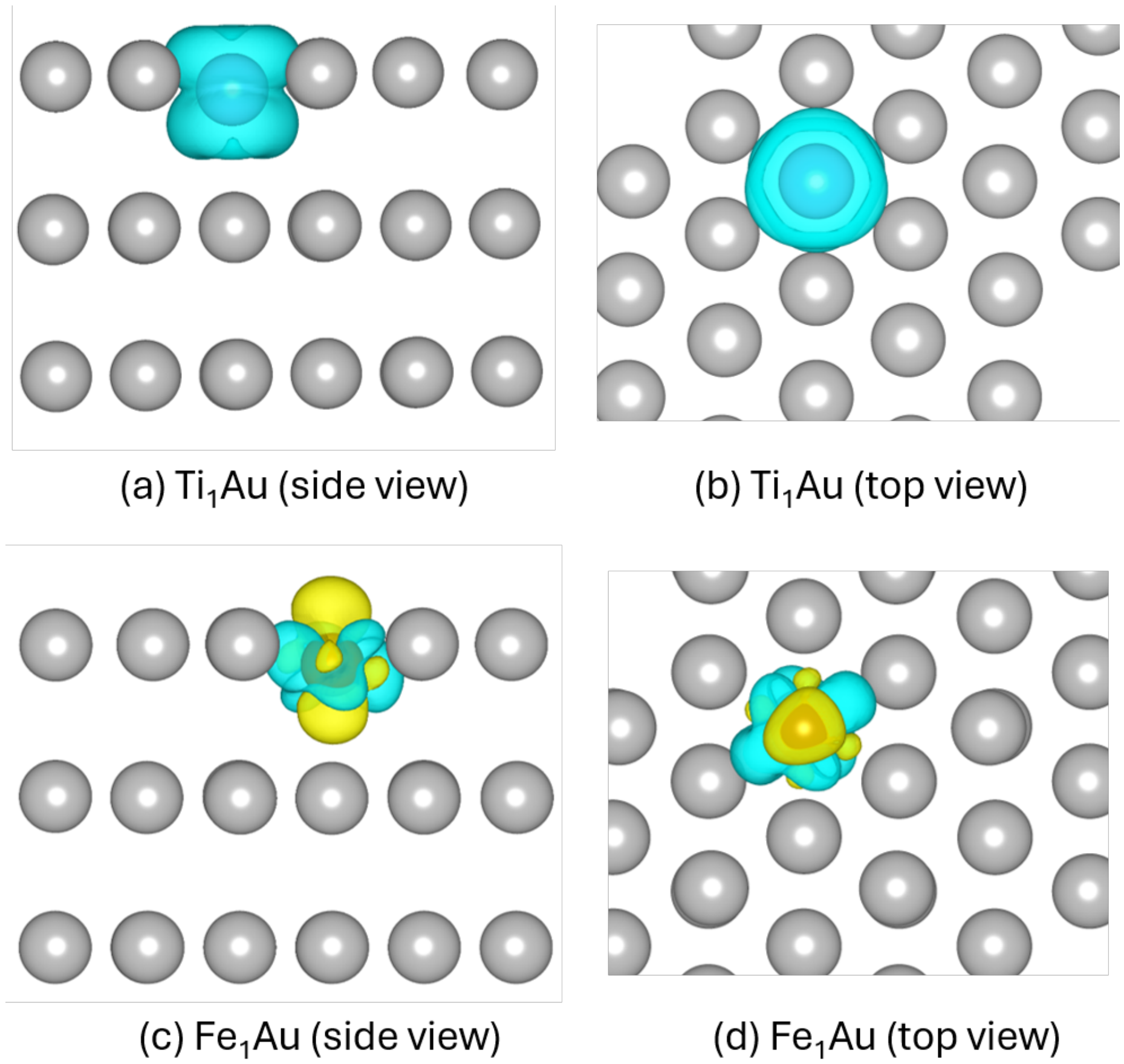

Figure S5. The real-space spin-density distributions of Ti_1Au and Fe_1Au.

Table S1. Change in adsorption energy (eV), d-band center (eV), and d-charge (e) for magnetic SAAs.

Surface	Dopant	ΔE_{ads}	$\Delta \varepsilon_d$	Δq_d
Ag	Co	-0.130	1.342	-0.019
Ag	Cr	-0.317	0.773	-0.117
Ag	Fe	-0.217	1.576	0.169
Ag	Mn	-0.325	2.208	0.095
Ag	Mo	-0.150	0.642	0.05
Ag	Re	-0.071	0.590	0.037
Ag	Ta	-0.004	0.253	-0.017
Ag	Tc	-0.12	0.035	0.095
Ag	Ti	-0.078	0.198	-0.046
Ag	V	-0.142	0.265	0.051
Ag	W	-0.212	0.182	-0.04
Ag	Nb	-0.070	0.064	-0.097
Au	Co	-0.156	0.783	
Au	Cr	-0.183	0.449	
Au	Fe	-0.185	1.497	
Au	Mn	-0.236	1.513	
Au	Mo	-0.066	0.244	
Au	Re	-0.049	0.305	
Au	Ru	-0.02	-0.059	
Au	Tc	-0.12	0.08	
Au	Ti	-0.02	0.158	
Au	V	-0.068	0.114	
Au	W	-0.083	0.303	
Cu	Co	-0.047	0.371	

Cu	Cr	-0.163	0.561	
Cu	Fe	-0.184	1.129	
Cu	Mn	-0.263	1.35	
Cu	Tc	-0.02	0.153	
Cu	V	-0.059	0.257	

Table S2. CO adsorption energy (eV) on representative magnetic SAAs.

	Co_1Ag	Co_1Au	Fe_1Ag	Fe_1Au	Mn_1Ag	Mn_1Au
spin	-2.29	-1.94	-1.63	-1.52	-1.21	-0.92
non-spin	-2.53	-2.28	-2.67	-2.38	-2.64	-2.47
ΔE(spin-nonspin)	0.24	0.34	1.04	0.86	1.44	1.55

Additional Methodological Information

Table S3. Impact of spin-orbit coupling (SOC) on magnetic moments (μ_B).

	Mn_1Au	Fe_1Au	Co_1Au
No SOC	4.56	3.12	1.74
SOC	4.54	3.12	1.71

Table S4. H adsorption energy (eV, relative to gas-phase H) on Co_1Ag, Fe_1Au and Mn_1Ag, using 4-layer model, 6-layer model, and 4-layer model with dipole correction.

	Co_1Ag	Fe_1Au	Mn_1Ag
4 layer	-2.61	-2.32	-2.41
6 layer	-2.59	-2.31	-2.42
4 layer with dipole correction	-2.61	-2.32	-2.40
